# Within-Person Fluctuations in Ethnic–Racial Affect and Discrimination-Based Stress: Moderation by Average Ethnic–Racial Affect and Stress

**DOI:** 10.1002/jad.12484

**Published:** 2025-02-16

**Authors:** Carolina Gonçalves, Dian Yu, Natasha Keces, Richard M. Lerner

**Affiliations:** 1 Department of Child Study and Human Development, Tufts University, Medford, Massachusetts, USA; 2 Department of Health Policy, Vanderbilt University, Nashville, Tennessee, USA; 3 Department of Psychology, Oklahoma State University, Stillwater, Oklahoma, USA

**Keywords:** discrimination, ethnic–racial identity, person-specificity, positive affect, stress, within-person fluctuations

## Abstract

**Introduction:**

Despite evidence highlighting the dynamic nature of ethnic–racial identity (ERI) development and the common occurrence of discriminatory experiences, many studies treat these constructs as static and equivalent across individuals. Drawing upon the Phenomenological Variant of Ecological Systems Theory (PVEST), this study examined the within-person covariations between ethnic–racial affect (individuals' positive feelings regarding their ethnic–racial background) and discrimination-based stress, and whether these relations were moderated by average affect and average stress.

**Method:**

This study employed an intensive longitudinal design with 771 observations nested within 133 participants (Mage=16.07, SD = 0.67), 52.3% were girls and ~93.3% were African American from Chicago, Illinois.

**Results:**

Results from the multilevel model analysis revealed that within-person fluctuations in ethnic–racial affect were predicted by discrimination-based stress and that these fluctuations were person-specific. Furthermore, findings from this study also showed that the within-person fluctuations in ethnic–racial affect in relation to stress from discrimination were weaker for those with higher average affect and stronger for those with higher average stress.

**Conclusions:**

This study highlights the dynamic and situational nature of developmental processes by emphasizing the within-person fluctuations and person-specificity. These findings highlight the importance of developing and delivering interventions and programs that promote positive ethnic–racial affect to mitigate the negative impact of discrimination. These initiatives should be offered consistently and tailored to address individuals' specific needs to maximize their effectiveness.

## 1 ∣ Introduction

Ethnic and racial identity (ERI) is considered a normative developmental task in which all youth are expected to engage as part of the identity formation process, particularly in the United States of America (Rivas-Drake and Umaña-Taylor 2019; Williams et al. 2012). ERI is defined as the process and content of developing an understanding and sense of belonging regarding one's ethnic and/or racial group (Umaña-Taylor 2011). This multifaceted and complex construct encompasses several dimensions including exploration (the degree to which individuals have engaged in activities to learn more about their ethnic–racial background); resolution (the extent to which individuals have gained clarity regarding the meaning of race and ethnicity to their sense of self); affirmation (individuals' feelings and attachment to their ethnic–racial group); centrality (how important individuals' racial identity is to their sense of self); and private/public regard (the positive or negative feelings individuals have/believe others have toward their racial group; Cross and Vandiver 2001; Phinney 1989, 2000; Sellers et al. 1998; Umaña-Taylor et al. 2004, 2014).

Components of ERI process (e.g., exploration and resolution) have been linked to positive outcomes, especially among ethnic and racial minoritized adolescents including better well-being, self-esteem, social functioning, academic achievement, and fewer externalizing behaviors, internalizing problems, and depressive symptoms (Rivas-Drake, Seaton, et al. 2014; Rivas-Drake, Syed, et al. 2014). Moreover, ethnic–racial affect, which encompasses affirmation and private regard, has been identified as a component that commonly emerges as a cultural asset that may offer protection against maladjustment (Rivas-Drake, Seaton, et al. 2014; Rivas-Drake, Syed, et al. 2014). For instance, findings from Brittian et al. (2015) highlighted ERI ethnic affirmation as a cultural factor that may reduce the potential risk of ethnic group discrimination on Latino young adults' mental health. Furthermore, social identity theory emphasizes the critical role of positive ethnic–racial affect in the psychological adjustment of ethnic and racial groups (Tajfel and Turner 1986). Building on this foundation, major contemporary theories of ERI have identified positive ethnic–racial affect as a central dimension of youth ERI development (Cross 1991; Sellers et al. 1998; Umaña-Taylor et al. 2004). Consequently, there is a need for further investigation into its variability and its associations with the effects of discrimination (negative attitudes and unfair treatment toward members of a specific group; Chin et al. 2020).

Ethnic–racial affect may exhibit greater variability and undergo more frequent changes during adolescence compared to other ERI components due to its unique association with cognitive processes (feelings and attitudes) that are especially malleable during this time (Sarsar et al. 2023). Furthermore, these fluctuations may be person-specific as some adolescents exhibit higher levels of ethnic–racial affect that protect them against the negative effects of discrimination, whereas others may experience significant fluctuations in experiences of discrimination due to their intersectional marginalized identities (Velez and Spencer 2018). Nevertheless, very few studies have explored within-person fluctuations in ethnic–racial affect with discrimination-based stress and whether these associations are person-specific, meaning that the ways individuals experience and respond to these phenomena vary depending on their unique personal histories, social contexts, and intersectional identities (Bornstein 2019; Molenaar 2013; Velez and Spencer 2018).

Understanding how adolescents' ethnic–racial affect fluctuates over time in relation to discrimination-based stress may provide important insights into the immediate coping strategies that adolescents use in the face of discrimination. By capturing within-person fluctuations and examining whether they are person-specific, researchers can also (1) identify specific times or conditions under which specific adolescents experience shifts in ethnic–racial affect and (2) investigate the protective role of ethnic–racial affect in buffering against stress from discrimination. Such information can guide the development of targeted interventions that are responsive to varying levels of ethnic–racial affect and stress. Therefore, this study examined how ethnic–racial affect varies within individuals and how these potential variations coact with weekly stress from discrimination. This study also examined whether average levels of ethnic–racial affect and average levels of discrimination-based stress moderate the within-person relations between fluctuations in ethnic–racial affect and stress from discrimination.

## 2 ∣ Theoretical Framework

Dynamic, relational, and developmental systems-based theoretical perspectives have situated human development, including identity formation, within the specific context of youth while, at the same time, emphasizing fluctuations and variability that occur in developmental processes (Bronfenbrenner and Morris 2006; Lerner 2018; Overton 2015). The Phenomenological Variant of Ecological Systems Theory (PVEST) is a human development framework that illustrates the impact of feedback from the environment on an individual's self-organization (Spencer 2006, 2024; Spencer et al. 1997). In addition, PVEST emphasizes experiences unique to the individual within a broader ecological context which may include connections between risks, stressors, coping responses, identity processes, and outcomes (Spencer et al. 1997). Accordingly, identity development in response to systematic stress and risks is person-specific, implying that there is no singularly applicable path to a positive identity for all individuals. Therefore, studies should focus on the heterogeneity in developmental pathways as compared to focusing primarily on an average pattern.

PVEST is an appropriate model for this study because it also argues that, although discrimination may be a pervasive stressor, children and youth of color have support systems and coping strategies that mitigate the effects of ethnic–racial discrimination (Spencer et al. 1997, 2015). As a salient component of youth development especially for youth of color, ethnic–racial affect may undergo several reorganizations based on adolescents' experiences within their context and may also serve as a coping strategy. Given its emphasis on the uniqueness of everyone's experiences within a broader ecological context, PVEST stresses the importance of within-person fluctuations and person-specificity in different developmental domains such as ERI (Spencer 2006, 2024; Spencer et al. 1997). Understanding developmental changes such as ethnic–racial affect during adolescence may be limited by the usage of one static score to represent a highly salient and complex component of identity development.

## 3 ∣ Within-Person Fluctuations in Ethnic–Racial Affect

Adolescence is marked by important social and cognitive transitions related to abstract thinking, formal reasoning, increased autonomy, and independence (Steinberg and Morris 2001). These changes coact with environmental factors and vary across individuals given the uniqueness of their environment. For adolescents from racial and ethnic minoritized backgrounds, navigating experiences related to racism and discrimination (Seaton and Iida 2019), along with these social and cognitive transitions may further complicate ERI development.

As a dynamic and multifaceted process, ERI development, especially among minoritized adolescents, must be captured through longitudinal methods that allow for the examination of within-person fluctuations over time and how contextual factors such as stress influence changes in identity development. For instance, Kiang et al. (2010) examined adolescents' ERI (affirmation and exploration) once over 4 years using within-person analyses and found that individuals exhibited substantial fluctuations in ERI across the years. Therefore, this study emphasizes the necessity of examining within-person fluctuations in ERI processes using repeated observations, such as intensive longitudinal methods (i.e., involving daily or at least weekly measurements). Furthermore, these fluctuations must be examined with constructs that are directly linked to ERI development and salient during this time such as stress from discrimination (Seaton and Iida 2019; Yip 2018).

By focusing on within-person fluctuations, researchers can better capture how adolescents' feelings regarding their ethnic–racial group may serve as protective factors against the negative effects of racism and discrimination. This approach allows for a more nuanced understanding of the interplay between individual development and contextual influences, ultimately informing strategies to support the well-being of these adolescents. Nevertheless, relatively few studies have assessed ERI longitudinally (for exceptions: Kiang et al. 2010; Torres and Ong 2010) and even fewer have examined short-term within-person fluctuations in the associations between ethnic–racial affect and discrimination-based stress.

### 3.1 ∣ Ethnic–Racial Affect and Discrimination-Based Stress

Discrimination has been identified as a significant threat and a chronic stressor that disproportionately affects individuals (Neblett et al. 2012; Williams and Mohammed 2009). Using a daily-diary method, findings from Seaton and Douglass (2014) revealed that African American youth reported a daily average of 2.4 racially discriminatory events and that the daily perceptions of racial discrimination were linked to depressive symptoms on the following day. Furthermore, studies have shown that a pathway by which discrimination may influence psychological well-being is through discrimination-based stress, given the associations between discrimination and the autonomic nervous system and hypothalamic–pituitary–adrenal axis activities, which are two major stress response systems (Lanier et al. 2017; Nam et al. 2022; Seaton et al. 2011).

Most empirical work examining relations between ERI components and effects from discrimination uses cross-sectional methods or focuses primarily on exploration and resolution (Romero and Roberts 2003; Umaña-Taylor and Updegraff 2007). As an exception, Zeiders et al. (2019) examined within-person changes in discrimination and components of ERI (exploration, resolution, and affirmation) among a sample of Mexican-origin adolescents yearly over 6 years. Results from this study revealed that within-person changes in discrimination predicted subsequent ERI resolution and affirmation with increases in discrimination within an individual predicting a decrease in ERI resolution and affirmation 1 year later (Zeiders et al. 2019). Although this study illuminates the importance of examining within-person fluctuations, the findings are inconsistent with previous studies and theories that have suggested that ERI dimensions can buffer against the negative effects of discrimination (Romero and Roberts 2003; Brittian et al. 2015; Umaña-Taylor et al. 2014).

According to the identification-attribution model, as individuals' ERI develops, they may become increasingly aware of discrimination (Gonzales-Backen et al. 2018). In response to this increased awareness, individuals may seek ways to cope with these experiences, that may involve drawing on their ERI as a source of support. The process of relying on ERI as a coping mechanism is aligned with the rejection-identification model. Rooted in social identity theory, the rejection-identification model posits that experiences of discrimination precede ERI formation and may lead to further ERI formation (Gonzales-Backen et al. 2018; Tajfel and Turner 1986). Given the immediate impact and common occurrence of discrimination, it is possible that using a more intensive longitudinal method (weekly vs. yearly) may help clarify the inconsistent findings between ERI and some specific effects of discrimination. Therefore, frequent assessments of adolescents' ethnic–racial affect in response to discrimination-based stress allow for a more accurate understanding of how adolescents may use positive feelings regarding their ethnic–racial groups to cope with the immediate effects of discrimination.

### 3.2 ∣ Moderating Effects of Average Ethnic–Racial Affect and Average Stress

By emphasizing the dynamic exchanges between the individual and their environment, PVEST also highlights the need for studies that focus on understanding person-specific characteristics—such as average levels of ethnic–racial affect and discrimination-based stress—that may moderate the within-person covariations between ethnic–racial affect and discrimination-based stress (Spencer 2006; Spencer et al. 1997, 2015). Several studies have examined the role of ERI as a moderator in the associations between ethnic–racial discrimination and health outcomes with findings revealing that different components of ERI can buffer against the negative effects of ethnic–racial discrimination, including stress (Woo et al. 2019; Yip et al. 2020). However, these studies mainly use cross-sectional methods. Furthermore, average levels of ethnic–racial affect or discrimination-based stress are frequently overlooked as potential moderators in research.

It is imperative to examine the extent to which average levels of ethnic–racial affect or average stress may impact the within-person covariations between ethnic–racial affect and discrimination-based stress. This examination may help identify conditions under which ethnic–racial affect serves as a protective factor and situations where discrimination-based stress amplifies the vulnerability to fluctuations in ethnic–racial affect. Such findings would deepen our understanding of the dynamic and individualized ways that adolescents navigate identity and stress within a broader social context marked by systemic challenges (Spencer et al. 1997).

## 4 ∣ The Current Study

Using an intensive longitudinal design, this study examined the within-person fluctuations between weekly ethnic–racial affect and discrimination-based stress among minoritized adolescents. The following research questions were examined (1) Do within-person fluctuations in discrimination-based stress predict fluctuations in ethnic–racial affect? (2) Are the aforementioned relations heterogenous/person-specific? (3) Does average ethnic–racial affect moderate the within-person relations between ethnic–racial affect and stress from discrimination? And lastly, (4) does average discrimination-based stress moderate the within-person relations between ethnic–racial affect and stress? Given the protective role of ERI, we hypothesized that higher-than-usual discrimination-based stress would predict elevated ethnic–racial affect and that these fluctuations would be person-specific. We also hypothesized that these covariations would be stronger for individuals with higher averages of ethnic–racial affect and discrimination-based stress, considering that sustained levels of positive feelings regarding one's ethnic–racial group may enhance resilience by reinforcing positive self-concept, whereas higher chronic stress levels may intensify sensitivity to weekly fluctuations in identity-related stressors (Butler-Barnes et al. 2017; Douglass and Umaña-Taylor 2017).

We addressed the research questions using a multilevel modeling (MLM) framework. An MLM framework is particularly useful in separating the within-person (Level 1) and between-person (Level 2) effects. The inclusion of a random slope allowed the within-person relations between ethnic–racial affect and stress to be different for different people, reflecting person-specificity. To separate the within-person and between-person effects, we also group mean-centered within-person predictors. Thus, within-person fluctuations were only compared to individual average levels, also reflecting a person-specific comparison.

## 5 ∣ Methods

This study was part of a larger study that explored individual components of developmental trajectories among students using multiple constructs. All study procedures were approved by the Institutional Review Board at Tufts University.

### 5.1 ∣ Participants and Procedures

Participants were recruited from a high school in Chicago, Illinois, in the United States that serves predominantly Black students. Both participants' assent and parental consent were obtained before data collection. Participants responded to surveys with questions and online tasks every Friday for 16 consecutive weeks. Originally, 204 participants were recruited in this study. Weekly surveys without ERI responses were excluded as invalid responses. Participants with fewer than three valid responses were excluded (N=35) to avoid estimation problems for random slopes. We also excluded participants with minimum within-person ERI variance (within-person SD < 5; N=36). Those who were excluded and included in the analytical sample did not significantly differ in gender ($\chi^{2}(1)=0.38$, *p* = 0.54), age (t=1.49, *p* = 0.14), average ERI level (t=−1.23, *p* = 0.22), or average discrimination-based stress (t=0.28, *p* = 0.78).

The final analytical sample included 771 observations nested within 133 participants. On average, each participant had 5.80 valid responses (SD = 2.03, range = 2–10). The students were between 13.42 and 18.83 years of age (M=16.02, SD = 0.67). There were 66 girls (50.3%), and 95.4% of the participants identified as Black/African American. Approximately 19.8% of parents reported having earned at least a 4-year college degree, 50.4% had a high school degree or GED or some college education, and 29.8% had less than a high school degree.

### 5.2 ∣ Measures

Given that this study involved intensive data collection using multiple measures, only three items were used for ethnic–racial affect and one item for discrimination-based stress to reduce participant burden. The items for ethnic–racial affect were adapted from the Ethnic Identity Scale (EIS; Umaña-Taylor et al. 2004) with some wording modified for clarity. These items included the following, “I have been happy that I am a member of my ethnic/racial group,” “I have had a strong sense of belonging to my own ethnic/racial group,” and “I have felt good about people from my ethnic/racial group.” Average scores from these items were used to indicate overall ethnic–racial affect with higher scores indicating more positive ethnic–racial affect. The original items from the EIS measure were developed to capture between-person differences and may not be sensitive to within-person change. Therefore, we changed the scale to 1 (*No Agreement*)–100 (*Complete Agreement*) to capture more nuanced within-person variances. Numbers closer to 1 indicate less positive feelings about one's ethnic–racial group.

Discrimination-based stress was assessed using an item from the stress measure by Almeida (2005) and Koffer et al. (2016). The item stated: “Today, I felt upset about being discriminated against because of my race, ethnicity, or gender.” The limitations of using this item will be discussed. Participants responded on a slider scale of 1–100 with higher scores indicating higher levels of stress from discrimination.

### 5.3 ∣ Analytic Strategy

Before addressing the research questions, preliminary analyses were conducted. All analyses were performed in R using packages *lme4* (Bates et al. 2015; Posit Team 2023) and *multilevelTools* (Geldhof et al. 2014). The within-level ($\omega^{w}$) and between-level ($\omega^{b}$) reliability indices were calculated for ethnic–racial affect items (Geldhof et al. 2014). For the within-level reliability ($\omega^{w}$) we found a reliability estimate of 0.636 with a 95% CI of [0.593, 0.680]. These results indicated that ~63% of the variance in the within-person part of the scale scores was accounted for by the common factor of ethnic–racial affect. For the between-level reliability ($\omega^{b}$) we found a reliability estimate of 0.993 with a 95% CI of [0.986, 1]. These results indicated that the common factor of ethnic–racial affect accounted for ~99% of the variance in the between-person part of the scale scores. Personal mean and SD across all responses were computed for each participant to examine whether we captured within-person variabilities in ethnic–racial affect and discrimination-based stress. Intraclass correlations were then computed to partition the variance at between- and within-person levels. MLM was conducted to answer the research questions.

A random intercept fixed slope model revealed that age and gender did not predict ethnic–racial affect, but response timepoint and total number of response occasions were linked to ethnic–racial affect. Thus, timepoint and occasions were included as covariates in all multilevel models. Timepoint was also included to control for potential measurement reactivity (changes in responses due to repeated exposure to the survey), and total occasions were also included to control for between-person differences based on different levels of engagement. Personal average discrimination-based stress was included to account for the between-person relations between ethnic–racial affect and stress from discrimination.

For Level 1/within-person predictors, weekly discrimination-based stress was centered using personal means. Cluster-mean centering helps separate within-person and between-person effects. This way, we only compare individuals to their personal means, reflecting a *person-specific* comparison. Timepoint was centered at 1, so 0 reflects the first measurement occasion. Regarding Level 2/between-person predictors, personal average discrimination-based stress was centered using the average across personal means. The number of total occasions was centered using means across individuals. Confidence intervals were computed for all model coefficients.

To answer the first three research questions, we conducted two multilevel models: a fixed slope model and a random slope model. The multilevel model equations are listed below:

#### 5.3.1 ∣ Model 1—Fixed Slope Model Equation


Level 1: $$ERI_{ij}=\beta_{0i}+\beta_{1}{\left(\text{stress}\right)}_{ij}+\beta_{2}{\left(\text{timepoint}\right)}_{ij}+r_{ij}\;\;r_{ij}∼N(0,\sigma^{2}).$$

Level 2: $$\beta_{0i}=\gamma_{00}+\gamma_{01}{\left(\text{occasion}\right)}_{i}+\gamma_{02}{\left(\text{stress}\_M\right)}_{i}+\mu_{0i}\;\;\mu_{0i}∼N(0,\tau_{00}).$$

Combined: $$ERI_{ij}=\gamma_{00}+\gamma_{01}{\left(\text{occasion}\right)}_{i}+\gamma_{02}{\left(\text{stress}\_M\right)}_{i}+\beta_{1}{\left(\text{stress}\right)}_{ij}\;\;+\beta_{2}{\left(\text{time}\right)}_{ij}+\mu_{0i}+r_{ij}.$$

#### 5.3.2 ∣ Model 2—Random Slope Model Equation


Level 1: $$ERI_{ij}=\beta_{0i}+\beta_{1i}{\left(\text{stress}\right)}_{ij}+\beta_{2}{\left(\text{timepoint}\right)}_{ij}+r_{ij}\;\;r_{ij}∼N(0,\sigma^{2}).$$

Level 2: $$\beta_{0i}=\gamma_{00}+\gamma_{01}{\left(\text{occasion}\right)}_{i}+\gamma_{02}{\left(\text{stress}\_M\right)}_{i}+\mu_{0i}\left[\begin{array}{c} \mu_{0i}\\ \mu_{1i} \end{array}\right]∼N\left(\left[\begin{array}{c} 0\\ 0 \end{array}\right],\left[\begin{array}{cc} \tau_{00} & \\ \tau_{10} & \tau_{11} \end{array}\right]\right).\;\;\beta_{1i}=\gamma_{10}+\mu_{1i}.$$

Combined: $$ERI_{ij}=\gamma_{00}+\gamma_{01}{\left(\text{occasion}\right)}_{i}+\gamma_{02}{\left(\text{stress}\_M\right)}_{i}+\gamma_{10}{\left(\text{stress}\right)}_{ij}\;\;+\beta_{2}{\left(\text{timepoint}\right)}_{ij}+\mu_{1i}{\left(\text{stress}\right)}_{ij}+\mu_{0i}+r_{ij}.$$

Response j is nested within individual i. In Model 1, a random intercept was included to account for between-person differences in ethnic–racial affect. $\tau_{00}$ is the residual variance of random intercepts and $\sigma^{2}$ is the within-person residual variance in ethnic–racial affect. $\beta_{1}$ is the fixed within-person effect of weekly stress on ERI. Building upon Model 1, Model 2 included a random slope for weekly discrimination-based stress. The random slope represents the person-specificity in the within-person relation. $\gamma_{10}$ is the average slope of weekly stress. $\tau_{11}$ is the between-person variance of the random slope. $\tau_{10}$ is the covariance between the residual of random intercept and random slope.

Regarding the *first research question* on the within-person relations between ethnic–racial affect and discrimination-based stress, $\beta_{1}$ in Model 1 and $\gamma_{10}$ in Model 2 both indicate the average within-person relations. To answer the *second research question* on heterogeneity/person-specificity, we conducted a likelihood ratio test to determine whether including a random slope fits the data better. If Model 2 demonstrated a significantly better fit with the data, the results support that the within-person relation is person-specific. To answer the *third research question*, we interpreted the covariance between the residual of random intercept and random slope in Model 2. Random intercepts in Model 2 indicated the personal average level of ethnic–racial affect after controlling for the total number of occasions and a personal average level of discrimination-based stress. The covariance between the residual of the random intercept and random slope can be interpreted as how the personal average level of ERI can alter the within-person associations between ethnic–racial affect and stress.

To answer the *fourth research question*, we added personal average discrimination-based stress as a predictor for the random slope to Model 2. The multilevel model equations are listed below:

#### 5.3.3 ∣ Model 3—Cross-Level Interaction


Level 1: $$ERI_{ij}=\beta_{0i}+\beta_{1i}{\left(\text{stress}\right)}_{ij}+\beta_{2}{\left(\text{timepoint}\right)}_{ij}+r_{ij}\;\;r_{ij}∼N(0,\sigma^{2}).$$

Level 2: $$\beta_{0i}=\gamma_{00}+\gamma_{01}{\left(\text{occasion}\right)}_{i}+\gamma_{02}{\left(\text{stress}\_M\right)}_{i}+\mu_{0i}\left[\begin{array}{c} \mu_{0i}\\ \mu_{1i} \end{array}\right]∼N\left(\left[\begin{array}{c} 0\\ 0 \end{array}\right],\left[\begin{array}{cc} \tau_{00} & \\ \tau_{10} & \tau_{11} \end{array}\right]\right).\;\;\beta_{1i}=\gamma_{10}+\gamma_{11}{\left(\text{stress}\_M\right)}_{i}+\mu_{1i}.$$

Combined: $$ERI_{ij}=\gamma_{00}+\gamma_{01}{\left(\text{occasion}\right)}_{i}+\gamma_{02}{\left(\text{stress}\_M\right)}_{i}+\mu_{0i}\;\;+\gamma_{10}{\left(\text{stress}\right)}_{ij}+\gamma_{11}{\left(\text{stress}\_M\right)}_{I}\times {\left(\text{stress}\right)}_{ij}\;\;+\mu_{1i}{\text{stress}}_{ij}+\beta_{2}{\left(\text{time}\right)}_{ij}+r_{ij}.$$

$\gamma_{11}$ is the interaction effect between the personal average stress and weekly stress. With a significant interaction, we followed up with a simple slope analysis to examine the within-person associations given different personal average stress levels.

## 6 ∣ Results

Descriptive results of ethnic–racial affect and discrimination-based stress are presented in Table 1. We captured within-person fluctuations in both ethnic–racial affect (average SD = 10.76) and stress (average SD = 22.07). The *ICC* of ethnic–racial affect indicated that 54% of variance came from between-person differences and 46% of variance came from within-person fluctuations. The *ICC* of discrimination-based stress indicated that 23% of variance came from between-person differences and 77% of variance came from within-person fluctuations.

### 6.1 ∣ Within-Person Fluctuations in Ethnic–Racial Affect and Discrimination-Based Stress

To examine whether weekly discrimination-based stress predicted ethnic–racial affect, a fixed slope multilevel model and a random slope multilevel model were conducted. Both models yielded similar results for within-person relations (see Table 2). The fixed slope of weekly stress in Model 1 (β=0.07, 95% CI = [0.03, 0.11]) and the average slope of weekly stress in Model 2 (β=0.10, 95% CI = [0.04, 0.15]) indicated that, when participants experienced higher levels of stress related to discrimination compared to their personal average, they tended to report more positive feelings toward their ethnic–racial group.

### 6.2 ∣ Person-Specificity

**FIGURE 1 ∣:**
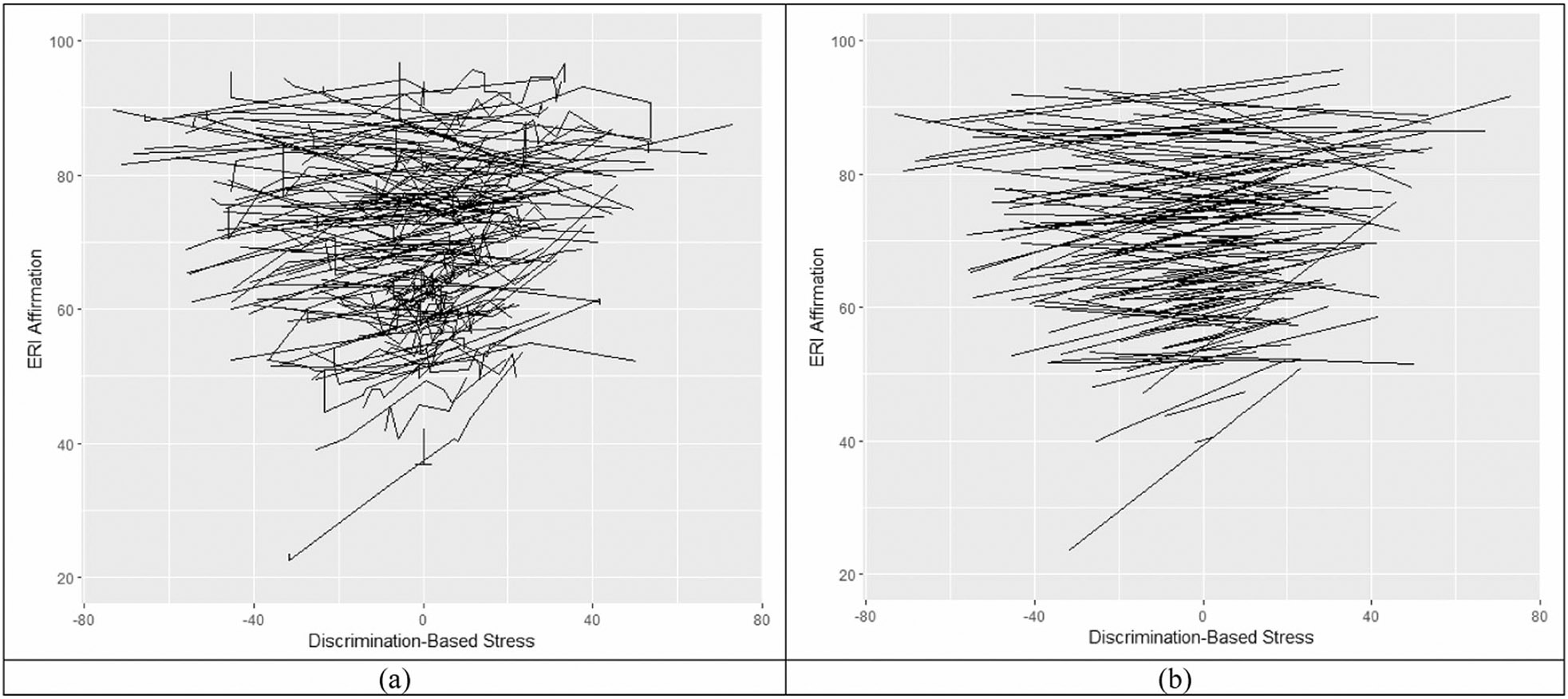
Spaghetti plots were generated based on (a) Model 2, and (b) a random slope model with group-centered stress as the only predictor to remove jaggedness caused by covariates. Models with and without covariates yielded similar results—the within-person slope is weaker for people with higher average ethnic–racial affect. Both graphs demonstrated substantial variabilities in the within-person relation between stress and ethnic–racial affect.

The likelihood ratio test revealed that the random slope model was significantly better than the fixed slope model ($\Delta \chi^{2}(2)=26.57$, *p* < 0.01). Allowing the within-person relations to vary across individuals fit the data better, implying that the within-person relations were person-specific. We generated a spaghetti plot of the relations between expected ethnic–racial affect and discrimination-based stress (Figure 1a). There are substantial variabilities of the slopes.

### 6.3 ∣ The Moderating Role of Average Ethnic–Racial Affect

There was a negative correlation (r=−0.50, 95% CI = [−0.74, −0.21]) between random intercept and random slope after controlling for number of occasions, and average discrimination-based stress was accounted for. This finding means that the within-person relations between weekly ethnic–racial affect and discrimination-based stress were weaker for people with higher average ethnic–racial affect (Figure 1b), as lines at the bottom of the graph appeared to be steeper than the lines at the top of the graph.

### 6.4 ∣ The Moderating Role of Average Discrimination-Based Stress

**FIGURE 2 ∣:**
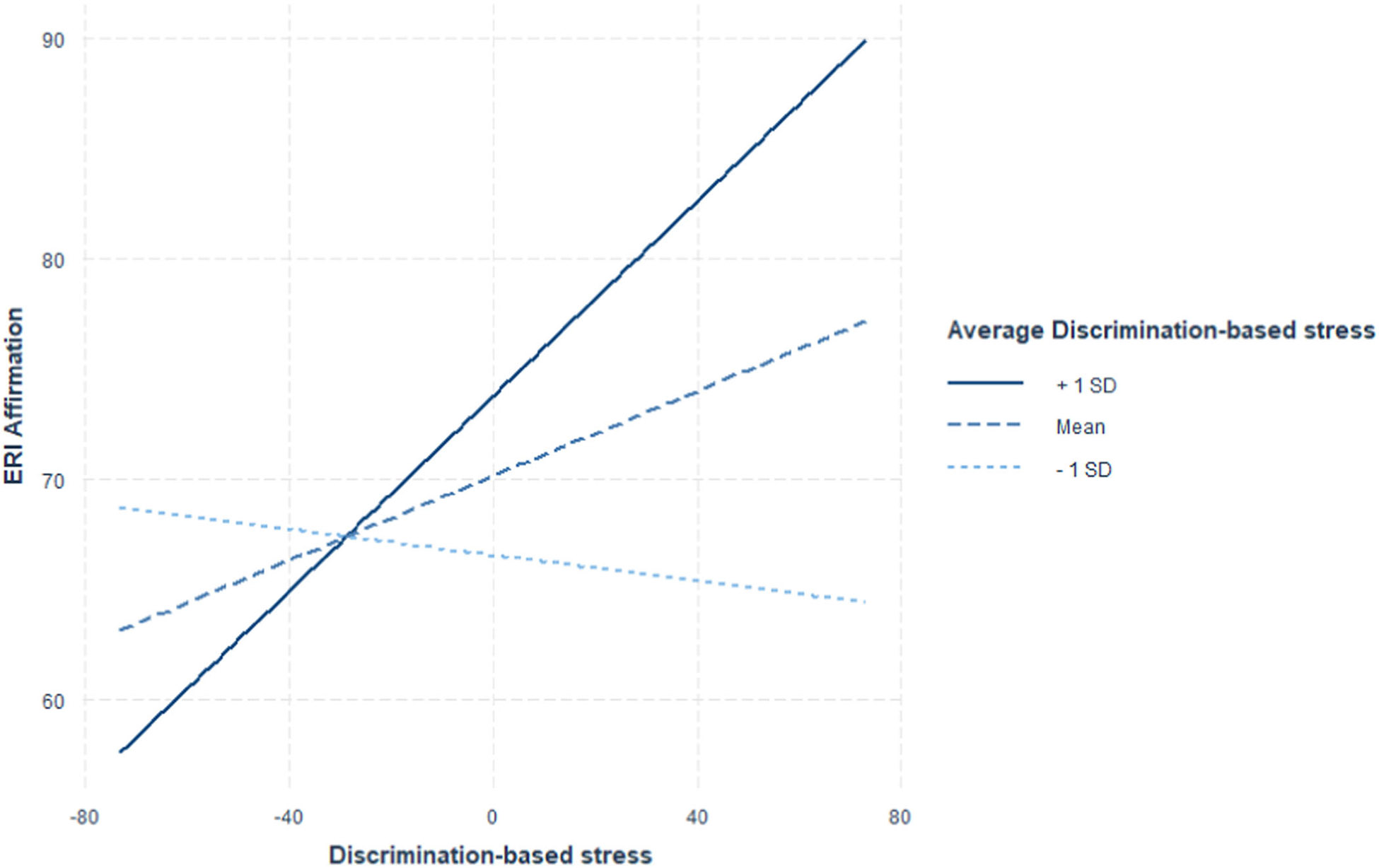
Simple slopes of discrimination-based stress on ethnic–racial affect (labeled as ERI affirmation) at 1 SD below mean, mean, and 1 SD above mean levels of average discrimination-based stress.

The cross-level interaction between average discrimination-based stress and weekly discrimination-based stress was tested in Model 3. The results showed a significant interaction (B=0.007, 95% CI = [0.004, 0.01]). With 10 points increase in average discrimination-based stress, the positive relation between ethnic–racial affect and stress increased by 0.07. Simple slope analysis further revealed that there was no association between ethnic–racial affect and discrimination-based stress when average perceived stress was 1 SD below the mean (β=−0.03, *p* = 0.44). The slope was significant when average discrimination-based stress was at the mean level (β=0.10, *p* < 0.01) and even stronger at 1 SD above the mean (β=0.22, *p* < 0.01). Such moderation is also captured in Figure 2 and Table 3. Overall, the within-person relations between ethnic–racial affect and stress were stronger for those who, on average, experienced higher discrimination-based stress.

## 7 ∣ Discussion

PVEST emphasizes the dynamic interrelations between individuals and their context and how experiences in an environment inform subsequent self-organizational processes (Spencer 2006, 2024; Spencer et al. 1997, 2015). Growing research on ERI development has identified the enactment of this process as an imperative task for minoritized youth (Umaña-Taylor 2011; Douglass and Umaña-Taylor 2017). The current study used an intensive longitudinal design to examine within-person fluctuations and weekly covariations between ethnic–racial affect and discrimination-based stress. Consistent with our hypotheses, this study found that experiencing higher-than-usual stress from discrimination is associated with higher ethnic-racial affect, and these findings were person-specific. However, the within-person associations between stress and ethnic–racial affect were weaker for those with higher average ethnic–racial affect and stronger for those with higher average stress, inconsistent with our hypothesis.

### 7.1 ∣ Within-Person Covariations in Ethnic–Racial Affect and Discrimination-Based Stress

We documented within-person fluctuations in ethnic–racial affect and discrimination-based stress, highlighting individual variability in these dynamic processes. These findings align with the theoretical frameworks that emphasize the dynamic nature of human development (Bronfenbrenner and Morris 2006; Overton 2015; Spencer 2006). Furthermore, we also found that weekly discrimination-based stress predicted increases in ethnic–racial affect. Therefore, when adolescents experienced higher than usual stress related to discrimination, they also reported more positive feelings and attitudes toward their ethnic–racial group. These results suggest that affirming one's connection to their ethnic–racial group could provide a sense of resilience that may buffer against the stress stemming from discriminatory experiences.

These findings are consistent with previous studies that have underscored the role of ERI as a protective factor and adaptive mechanism in the face of discrimination (Rivas-Drake, Seaton, et al. 2014; Rivas-Drake, Syed, et al. 2014). Furthermore, these findings also align with the rejection-identification model that highlights how individuals' increased awareness and experiences with discrimination may lead to reliance on one's identity for coping strategies (Gonzales-Backen et al. 2018). However, to our knowledge, no other studies have focused specifically on fluctuations of ethnic–racial affect and discrimination-based stress, nor used intensive longitudinal data to examine these associations. Therefore, this study provides a novel contribution to the literature by capturing the dynamic, within-person fluctuations in ethnic–racial affect in response to discrimination-based stress over time. This approach may offer a more nuanced understanding of the temporal relations between discrimination-based stress and ethnic–racial affect, highlighting the short-term adaptive processes that may otherwise be obscured in less frequent measurements.

### 7.2 ∣ Person-Specificity

Findings from this study also revealed that the within-person relations between ethnic–racial affect and discrimination-based stress were person-specific, suggesting that some adolescents may strengthen their positive feelings toward their ethnic–racial group in response to discrimination-based stress, whereas others may show negative or weaker responses. These processes operate in unique ways for everyone depending on their specific background, intersectional marginalized identities, and coping strategies. Therefore, it is imperative to consider individual variability rather than relying on group means to better understand adolescents' unique experiences and the processes they rely upon in the face of discrimination.

Person-specific approaches enable the identification of distinct profiles or subgroups of adolescents who may represent specific patterns of processes related to adolescent development (von Eye and Bogat 2006), including identity development and experiences of stress from discrimination. For instance, individuals demonstrating high levels of ethnic–racial affect and low levels of stress from discrimination may embody unique characteristics that are important to identify to better understand the protective factors that buffer against the adverse effects of discrimination. These individuals may provide critical insights into the role of positive ERI in fostering psychological well-being, navigating social stressors, and sustaining adaptive developmental trajectories (Meca et al. 2021). By studying such profiles, researchers can design targeted interventions that amplify protective factors while tailoring supports for youth who may experience heightened vulnerability due to differing patterns of ethnic–racial affect and discrimination-based stress. Therefore, these results emphasize the necessity of approaches that highlight the diversity or heterogeneity within a specific population, especially for ethnic–racial minoritized populations whose experiences of marginalization are often viewed as homogeneous.

### 7.3 ∣ The Moderating Role of Average Ethnic–Racial Affect and Average Stress

Within-person associations between weekly ethnic–racial affect and discrimination-based stress were weaker among adolescents with higher average ethnic–racial affect. This finding, which is inconsistent with our hypothesis, suggests that for adolescents who consistently exhibit positive feelings and attitudes toward their ethnic–racial group, the covariations between ethnic–racial affect and discrimination-based stress are less impactful. In turn, these covariations may be more pronounced for those with overall negative ethnic–racial affect. Therefore, consistent and stable ethnic–racial affect may reduce the intensity of weekly fluctuations in ethnic–racial affect and discrimination-based stress.

Furthermore, the within-person covariations between ethnic–racial affect and discrimination-based stress were stronger for adolescents with higher average discrimination-based stress. These findings may suggest that for adolescents who experience *higher overall* levels of stress due to ethnic–racial discrimination, each additional experience of discrimination may have a greater impact on their feelings and attitudes toward their ERI, leading to more noticeable increases in positive feelings regarding their ethnic–racial groups when stress from discrimination increase. These adolescents may be navigating cumulative experiences of discrimination due to additional marginalized identities including sexual orientation, immigrant status, gender, or religion. Therefore, it is possible that, in comparison to adolescents experiencing lower average stress related to discrimination, adolescents who on average experience higher stress related to discrimination may rely more on their positive feelings toward their ethnic–racial group to cope with the challenges of discrimination.

Overall, the moderating role of average ethnic–racial affect and average discrimination-based stress revealed the importance of a positive ethnic–racial affect and emphasized the heightened sensitivity toward ethnic–racial affect for those experiencing high levels of stress. Examining these moderating effects was imperative to identify for whom the associations between ethnic–racial affect and discrimination-based stress may be weaker or stronger. These findings underscore that a stable, positive identity can provide resilience against stress. Therefore, addressing these aspects of adolescent development, especially among minoritized adolescents, is crucial to support their well-being and development.

### 7.4 ∣ Strengths and Limitations

This study employed an intensive longitudinal method (weekly measurements) to capture within-person fluctuations in ethnic–racial affect and discrimination-based stress. This approach addresses a gap in the literature that often relies on cross-sectional designs to evaluate dynamic and complex processes related to human development. By focusing specifically on ethnic–racial affect, this study highlights that although collective ERI is beneficial to adolescents' overall development (Rivas-Drake, Seaton, et al. 2014; Rivas-Drake, Syed, et al. 2014), unique dimensions of ERI operate differently among individuals. Therefore, future studies should consider examining specific facets of ERI to fully understand their implications on youth outcomes, particularly in the face of discrimination.

The current study findings should be interpreted considering some limitations. First, our study relied on self-reported measures which are prone to issues of systematic error variance (Clifton 2020). Second, discrimination-based stress was assessed using a one-item measure from a stress measure with limited content validity. In addition, the description of the item assessed how “upset” an individual felt due to being discriminated against, which may arguably relate more to feelings of anger than stress. In addition, the description of the item included discrimination based on race, ethnicity, and gender despite the study's focus solely on race and ethnicity. Therefore, the discrimination-based stress measure serves as a major limitation for this study. More studies have used biomarkers of stress (e.g., cortisol levels, blood pressure, alpha amylase) to fully capture how discrimination impacts stress levels specifically among adults (Allen et al. 2019). Lastly, to reduce participant burden, we only used three items developed from the EIS measure which indicates that our findings cannot be compared to other findings that use the EIS measure.

In addition, the weekly survey completion rate was low, so it is possible that the final sample may not be representative of the full sample. Future studies should assess ways to increase the response rate for intensive longitudinal studies, especially among adolescents by including caregivers or educators to support data collection methods. Although the findings from this study may not generalize to all populations, they represent a valuable step forward in utilizing novel methods to research salient constructs among an understudied population.

Furthermore, the low weekly completion rate limited our ability to test potential bidirectional effects between ethnic–racial affect and discrimination-based stress. Lagged effects would be included in the model to test bidirectionality. However, such models require the spacing between observations to be even, and the low weekly completion rate resulted in a large number of missing time points in bidirectional models, leaving estimation untrustworthy. Hence, this study focused on the concurrent within-person associations between ethnic–racial affect and discrimination-based stress. Future intensive longitudinal studies with even time spacing and high completion rates should test the bidirectionality between these constructs.

### 7.5 ∣ Implications

The findings from this study highlight that adolescents' feelings and attitudes regarding their ethnic–racial group do not remain constant over time. Instead, these feelings undergo irregular and frequent changes in response to environmental factors including stress related to discrimination. Therefore, interventions or resources focused on heightening adolescents' positive feelings regarding their ethnic racial group should be implemented regularly, such as daily or weekly. For instance, receiving regular and constant positive messages regarding one's ethnic and racial group may increase individuals' ethnic–racial affect and result in longer-lasting positive effects. Furthermore, these interventions should be specifically tailored for each individual based on their unique background and experiences.

Although previous studies have also highlighted within-person fluctuations in discrimination (Zeiders et al. 2019), the current study provides more nuance by focusing on a key health-related effect from discriminatory experiences—stress—among a sample of predominantly Black adolescents. Focusing on stress from experiences of discrimination as opposed to just discrimination provides a more nuanced understanding of the long-lasting impact of discriminatory experiences on minoritized adolescents' health. Moreover, discrimination-based stress is linked to long-term poor health outcomes including higher depression and anxiety symptoms, poor self-esteem, and chronic disease (Nam et al. 2022; Seaton et al. 2011). Therefore, interventions aimed at reducing discrimination must focus on the long-term impact of these experiences to improve long-term health outcomes.

Furthermore, more policies should be designed to eliminate discrimination and its deleterious effects that target the sources of discrimination by identifying when adolescents' discrimination-based stress increases. For instance, educational policies should require schools to incorporate antidiscrimination content in their curricula, especially at the high school level, where students spend much of their time. One review study identified the need for systematic research on the important contributions of peers, caregivers, and educators for victims of discrimination, and the need for more evidence-based programming for adolescents (Fu et al. 2022). Findings from this review suggested that developing school-based microaggression antiracism programs aimed at decreasing discrimination is needed for minoritized and White youth (Fu et al. 2022). These policies should also include resources for individuals to cope with stress from discrimination that may include how to better rely on one's ethnic-racial group to increase one's feelings and attitudes toward this group.

The results from this study also highlighted the stabilizing effect of a strong baseline ethnic–racial affect and the heightened sensitivity of ethnic–racial affect among those experiencing high levels of stress related to discrimination. These results emphasize the need for future studies that examine common characteristics shared by adolescents for whom these findings apply to develop interventions and policies that support adolescents navigating stressful environments while reinforcing positive identity. These findings also imply that interventions and support for these adolescents should be context-specific and individualized to effectively promote positive identity development and mitigate the negative impacts of discrimination.

Within-person fluctuations in ethnic–racial affect and discrimination-based stress may occur in response to a myriad of contextual factors that are specific to that individual. By highlighting that these changes vary frequently for each person across time, this study emphasizes the need for more research to identify person-specific factors that could lead to an increase or decrease in adolescents' feelings about their ethnic and racial group. Similarly, stress from discrimination may also be higher for those with intersecting marginalized identities who may be more susceptible to negative health outcomes deriving from cumulative discriminatory experiences (Seng et al. 2012). This study elucidates the prolonged negative impact of experiences of discrimination, thus emphasizing that a single score is insufficient to identify the effects of discrimination on adolescent health. Therefore, within-person intensive longitudinal analysis allows researchers to obtain a more holistic understanding of key developmental assets during adolescence.

## 8 ∣ Conclusions

ERI is an important means by which ethnicity and race influence normative development and promote positive youth adjustment, especially for youth of color (Umaña-Taylor et al. 2014). This study focuses on one of the dimensions of ERI—ethnic–racial affect—to elucidate our understanding of the within-person fluctuations in ethnic–racial affect and discrimination-based stress. Overall, the findings from this study highlight the necessity for intensive longitudinal designs to better understand adolescent development, especially considering that biological, cognitive, and social changes that are prevalent during this time are most likely directly impacting fluctuations in ethnic–racial affect and discrimination-based stress.

## Figures and Tables

**TABLE 1 ∣:** Descriptives of personal means, SDs, range, and ICC of ERI and discrimination-based stress.

	Personal M		Personal SD		*ICC*
	M (SD)	Range	M (SD)	Range	
ERI	69.66 (13.52)	37.29–95.94	10.76 (5.47)	5.26–32.86	0.54
Stress	51.47 (17.54)	1.00–99.83	22.07 (12.72)	0.00–50.87	0.23

**TABLE 2 ∣:** Unstandardized coefficients of fixed slope and random slope models.

	Model 1		Model 2	
	*B*	95% CI	*B*	95% CI
Fixed effects				
Intercept	**71.76**	**[69.24, 74.27]**	**71.87**	**[69.36, 74.38]**
Level 1				
Timepoint	**−0.86**	**[−1.30, −0.42]**	**−0.91**	**[−1.34, −0.48]**
Weekly stress	**0.07**	**[0.03, 0.11]**	**0.10**	**[0.04, 0.15]**
Level 2				
Total occasions	1.08	[−0.08, 2.23]	**1.19**	**[0.08, 2.29]**
Personal mean stress	**0.21**	**[0.08, 0.34]**	**0.28**	**[0.15, 0.42]**
Random effects				
Intercept *residual variance*	150.70	**[114.34, 200.92]**	156.12	**[118.94, 207.64]**
Slope *variance*			0.04	**[0.02, 0.07]**
Intercept-slope correlation			−0.50	**[−0.74, −0.21]**
$\Delta \chi^{2}(df)$		26.57(*2*)**		

*Note:* Bolded coefficients indicate the 95% confidence interval does not contain zero.

** *p* < 0.01.

**TABLE 3 ∣:** Unstandardized coefficients of cross-level interaction model.

	Model 3	
	*B*	95% CI
Fixed effects		
Intercept	**71.88**	**[69.39, 74.37]**
Level 1		
Timepoint	**−0.91**	**[−1.22, −0.49]**
Weekly stress	**0.10**	**[0.04, 0.15]**
Level 2		
Total occasions	**1.17**	**[0.07, 2.28]**
Personal mean stress	**0.21**	**[0.08, 0.34]**
Stress × personal mean stress	**0.007**	**[0.004, 0.01]**
Random effects		
Intercept residual *variance*	**154.46**	**[118.00, 202.25]**
Slope residual *variance*	**0.03**	**[0.01, 0.05]**
Intercept-slope correlation	**−0.51**	**[−0.75, −0.21]**

*Note:* Bolded coefficients indicate the 95% confidence interval does not contain zero.

## Data Availability

The data that support the findings of this study are available on request from the corresponding author. The data are not publicly available due to privacy or ethical restrictions.
